# History of Infertility and Midlife Cardiovascular Health in Female Individuals

**DOI:** 10.1001/jamanetworkopen.2023.50424

**Published:** 2024-01-05

**Authors:** Amy R. Nichols, Sheryl L. Rifas-Shiman, Karen M. Switkowski, Mingyu Zhang, Jessica G. Young, Marie-France Hivert, Jorge E. Chavarro, Emily Oken

**Affiliations:** 1 Division of Chronic Disease Research Across the Lifecourse, Department of Population Medicine, Harvard Medical School and Harvard Pilgrim Health Care Institute, Boston, Massachusetts; 2 Department of Nutrition, Harvard T. H. Chan School of Public Health, Boston, Massachusetts; 3 Department of Epidemiology, Harvard T. H. Chan School of Public Health, Boston, Massachusetts; 4 Diabetes Unit, Massachusetts General Hospital, Boston

## Abstract

**Importance:**

Fertility status is a marker for future health, and infertility has been associated with risk for later cancer and diabetes, but associations with midlife cardiovascular health (CVH) in female individuals remain understudied.

**Objective:**

To evaluate the association of infertility history with CVH at midlife (approximately age 50 years) among parous individuals.

**Design, Setting, and Participants:**

Project Viva is a prospective cohort study of pregnant participants enrolled between 1999 and 2002 who delivered a singleton live birth in the greater Boston, Massachusetts, area. Infertility history was collected at a midlife visit between 2017 and 2021, approximately 18 years after enrollment. Data analysis was performed from January to June 2023.

**Exposures:**

The primary exposure was any lifetime history of infertility identified by self-report, medical record, diagnosis, or claims for infertility treatment.

**Main Outcomes and Measures:**

The American Heart Association’s Life’s Essential 8 (LE8) is a construct for ranking CVH that includes scores from 0 to 100 (higher scores denote better health status) in 4 behavioral (diet, physical activity, sleep, and smoking status) and 4 biomedical (body mass index, blood pressure, blood lipids, and glycemia) domains to form an overall assessment of CVH. Associations of a history of infertility (yes or no) with mean LE8 total, behavioral, biomedical, and blood biomarker (lipids and glycemia) scores were examined, adjusting for age at outcome (midlife visit), race and ethnicity, education, household income, age at menarche, and perceived body size at age 10 years.

**Results:**

Of 468 included participants (mean [SD] age at the midlife visit, 50.6 [5.3] years) with exposure and outcome data, 160 (34.2%) experienced any infertility. Mean (SD) LE8 scores were 76.3 (12.2) overall, 76.5 (13.4) for the behavioral domain, 76.0 (17.5) for the biomedical domain, and 78.9 (19.2) for the blood biomarkers subdomain. In adjusted models, the estimated overall LE8 score at midlife was 2.94 points lower (95% CI, −5.13 to −0.74 points), the biomedical score was 4.07 points lower (95% CI, −7.33 to −0.78 points), and the blood subdomain score was 5.98 points lower (95% CI, −9.71 to −2.26 points) among those with vs without history of infertility. The point estimate also was lower for the behavioral domain score (β = −1.81; 95% CI, −4.28 to 0.66), although the result was not statistically significant.

**Conclusions and Relevance:**

This cohort study of parous individuals found evidence for an association between a history of infertility and lower overall and biomedical CVH scores. Future study of enhanced cardiovascular preventive strategies among those who experience infertility is warranted.

## Key Points

**Question:**

Is infertility associated with midlife cardiovascular health (CVH) in female individuals?

**Findings:**

In this cohort study of 468 parous individuals, a history of infertility was significantly associated with lower CVH scores (range, 0-100) at midlife compared with participants with no infertility history. The overall CVH score was 2.94 points lower, the biomedical domain score was 4.07 points lower, and the blood metabolic biomarker subdomain score was 5.98 points lower among individuals with a history of infertility vs those without.

**Meaning:**

These findings suggest that a history of infertility may serve as a marker for future CVH in female individuals and provide important information for screening and prevention, potentially years before disease onset.

## Teaser

This cohort study evaluates the association of infertility history with cardiovascular health at midlife among parous individuals.

## Introduction

Physiological infertility—that is, the inability to achieve pregnancy after 12 or more months of unprotected intercourse (or ≥6 months if the individual is aged ≥35 years)^1,2^—affects approximately 15% of opposite-sex couples attempting conception.^3^ Lifetime risk estimates range from 2.6% to 31.8% worldwide,^4,5^ although these values are likely underestimated.^6^ The prevalence may be higher when considering uncoupled individuals or same-sex couples who do not meet the definitions for unassisted conception.^7^ Importantly, infertility does not imply sterility or childlessness; approximately one-half of female individuals who experience difficulties conceiving are parous,^8^ and one-third to one-half of couples meeting the clinical infertility definition subsequently conceive without medical interventions.^9,10^

The burden of infertility extends beyond involuntary childlessness and affects the overall physical, emotional, and social health of individuals and families.^6^ Outcomes may inequitably affect disadvantaged and marginalized populations owing to disparities in fertility care access and coverage.^11^ Increasing evidence indicates that reproductive traits may serve as markers for future cardiometabolic health.^12,13,14^ Advancing age is the primary factor associated with declining female fertility,^15^ particularly approaching age 40 years.^3^ Furthermore, early infertility may indicate underlying pathophysiology associated with later cardiometabolic outcomes.^16^ Conditions associated with infertility, including polycystic ovary syndrome (PCOS) and endometriosis, share underlying biological mechanisms with cardiovascular disease (CVD), including chronic inflammation and insulin resistance.^17,18,19^ Both infertility and CVD share common risk factors, including tobacco use, diet quality, and excess adiposity.^12,19^ Robust evidence of an association with infertility would provide an invaluable clinical marker to support cardiovascular health (CVH) screening and prevention efforts years before chronic disease manifests.

To address this important question, we used prospectively collected data from the Project Viva pregnancy cohort to evaluate the associations of a history of infertility with midlife CVH defined by the American Heart Association’s (AHA) Life’s Essential 8 (LE8), a validated composite score of CVH.^20^ To explore the potential for an observable association beyond known cardiovascular risk factors, we proposed models adjusted for age or for multiple cardiovascular markers (eg, body size, age, and socioeconomic status) not including behavioral indicators (eg, diet and smoking) incorporated into the LE8. We hypothesized that infertility would be associated with lower LE8 scores in parous individuals, particularly for infertility experienced before age 35 years.

## Methods

### Study Design and Cohort

As described elsewhere,^21,22^ Project Viva enrolled patients at less than 22 weeks’ gestation from 1999 to 2002 from Atrius Harvard Vanguard Medical Associates in Boston, Massachusetts. All participants had health insurance or Medicaid, resided within the greater metropolitan Boston area, and many were college educated. Data for this cohort study are based on enrollment characteristics and the midlife follow-up visit in 2017 to 2021, a mean of 18.3 years after the index delivery. Exclusion criteria included missing data for primary exposure or any LE8 domain prohibiting calculation of an aggregate score. A comparison of individuals included vs excluded is in eTable 1 in Supplement 1. All participants provided written informed consent at every visit; the Harvard Pilgrim Health Care institutional review board approved all study protocols. This study followed the Strengthening the Reporting of Observational Studies in Epidemiology (STROBE) reporting guidelines for cohort studies.

### History of Infertility

We derived history of infertility (yes vs no) from 3 data sources. First, at enrollment participants reported via questionnaire whether they were actively attempting to conceive and the number of menstrual cycles before conception. We defined infertility as 12 or more months, or 6 or more months if aged 35 years or older, of attempts to conceive.^1,3^ Second, we reviewed index pregnancy medical records and abstracted infertility diagnoses and claims for infertility services, including assisted reproductive technologies or fertility medication prescriptions (eg, clomiphene citrate, follitropin beta, or menotropin). Third, participants reported all past pregnancies by questionnaire at midlife, including months to conceive, assisted reproductive treatments, and a separate question about any diagnosed infertility.

### Cardiovascular Health

 The LE8 is an AHA construct encompassing 8 CVH components in 2 domains. First, the behavioral domain includes diet, physical activity, sleep health, and smoking status. Second, the biomedical domain includes weight (as body mass index [BMI], calculated as weight in kilograms divided by height in meters squared), blood pressure, blood lipids (non–high-density lipoprotein [HDL] cholesterol), and glycemia (fasting glucose or hemoglobin A_1c_ plus diabetes status).^20^ We also examined a blood biomarker subdomain (blood lipid and glycemia scores) for a granular examination of the biomedical domain.^20^ eTable 2 in Supplement 1 presents methods of measurement and quantification for each LE8 metric per Lloyd-Jones et al.^20^ We calculated the overall LE8 score and scores for each domain and subdomain on a scale from 0 to 100 points, with a higher score representing a healthier score, and averaged component scores for overall, behavioral, biomedical, and blood domains.^20^ An overall score of 80 to 100 is considered high, 50 to 79 moderate, and 0 to 49 low CVH; the national mean (SE) for female individuals aged 20 to 79 years is 68.1 (0.48) according to the National Health and Nutrition Examination Survey 2013 to 2018.^23^

### Covariates

To elucidate potential for overall association, as well as independent associations in the context of known CVD risk factors, we executed (1) models adjusted for age and (2) fully adjusted models of infertility status with midlife CVH. In fully adjusted models, we accounted for advancing age,^24,25^ earlier age at menarche,^26,27^ excess adiposity,^28,29,30,31^ and socioeconomic status^32,33^ measures including income and educational attainment.^20,34^ Items collected by questionnaire during early pregnancy included perceived body size at age 10 years, race and ethnicity (categorized as Hispanic, non-Hispanic Black, non-Hispanic White, and other, which includes American Indian or Alaska Native, non-Hispanic Asian or Pacific Islander, and participants who endorsed >1 race), education level, and annual household income. We included age at menarche reported approximately 13 years post partum and age at outcome approximately 18 years post partum. To assess body size before exposure, infertility, we used perceived body size at age 10 years (5 levels ranging from markedly underweight to markedly overweight); owing to small cell sizes, we collapsed the categories to underweight, average weight, and overweight. We included race and ethnicity in models as markers of social experiences, not biological differences, and because fertility treatment access may inequitably affect marginalized groups.^11^

### Statistical Analysis

Data analysis was performed from January to June 2023. We used multivariable linear regression to estimate associations between lifetime history of infertility (yes vs no) and continuous midlife LE8 scores. Because advancing age is the primary factor associated with female infertility and is an important characteristic associated with CVH, we adjusted all models for age at outcome (model 1), and then adjusted by the aforementioned covariate set (model 2). In addition, we were interested in examining LE8 score as a categorical outcome (high, ≥80; moderate, 50-79; and low, <49). Owing to small cell counts in the low category, we dichotomized LE8 as high vs low-to-moderate (reference, <80 points) in logistic regression models. For a comprehensive examination of the biomedical domain, secondary analyses examined (1) model 2 for each scored behavioral component as additional covariates and (2) each biomedical component as an outcome. Statistical significance was defined as α < .05. We conducted all analyses in Stata statistical software version 17.0 (StataCorp).

We used multiple imputation by chained equations^35^ to account for missing covariates. Models included 50 imputations among all participants, including all model covariates and potential factors associated with missingness. We report pooled estimates from the imputed data sets among the included participants with measured exposures and outcomes. These models met the assumptions underlying linear regression.

We conducted several sensitivity analyses. First, because female infertility at younger ages may indicate underlying physiological mechanisms related to CVH,^16,36,37^ we conducted analyses stratified by age at first instance of infertility (<35 vs ≥35 years). Second, we conducted subgroup analyses to gauge robustness of primary results by limiting our analyses to those with no known confounding biological mechanisms. We restricted the sample by excluding participants with PCOS diagnosis, those who experienced early-onset menopause (before age 45 years), and who were postmenopausal at outcome assessment.

## Results

 Of 2100 participants who delivered singleton live births, 703 completed the midlife visit, and 468 participants were included in the analysis (Figure). Included participants were more highly educated (college graduates, 344 of 468 participants [73.8%] vs 996 of 1632 participants [61.9%]), less likely to have ever smoked (125 of 468 participants [26.8%] vs 529 of 1632 participants [32.8%]), and had higher annual household income (>$70 000; 285 of 468 participants [66.4%] vs 839 of 1632 participants [59.1%]) than those who were excluded (eTable 1 in Supplement 1). Of the 468 included participants (mean [SD] age at the midlife visit, 50.6 [5.3] years), 160 (34.2%) had a history of infertility. Mean (SD) LE8 scores were 76.3 (12.2) overall, 76.5 (13.4) for the behavioral domain, 76.0 (17.5) for the biomedical domain, and 78.9 (19.2) for the blood biomarkers subdomain (Table 1). Those who experienced infertility were older at the midlife visit (mean [SD], 52.4 [4.8] vs 49.7 [5.3] years) and, at enrollment, were more likely to identify as non-Hispanic White (115 participants [71.9%] vs 197 participants [64.2%]), have attained a college degree (132 participants [82.5%] vs 212 participants [69.3%]), and have annual household income greater than $70 000 (114 participants [76.0%] vs 171 participants [61.3%]) compared with those who did not experience infertility. Both the biomedical domain and blood biomarker subdomain differed by infertility status (Table 1). Other characteristics did not differ meaningfully.

**Figure.**
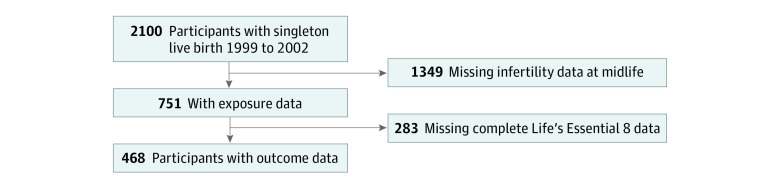
Study Sample Flowchart

**Table 1.** Participant Characteristics by Lifetime Infertility Status in Project Viva

Characteristic	Participants, No. (%)		
	Total (N = 468)	Lifetime history of infertility (n = 160 [34.2%])	No lifetime history of infertility (n = 308 [65.8%])
Infertility at enrollment	NA	90 (19.2)	NA
Age, mean (SD), y			
At menarche	12.8 (1.4)	12.7 (1.4)	12.8 (1.4)
At enrollment	32.5 (5.2)	34.3 (4.7)	31.5 (5.2)
At midlife visit	50.6 (5.3)	52.4 (4.8)	49.7 (5.3)
First infertility at age <35 y	NA	64 (40.0)	NA
First infertility at age ≥35 y	NA	94 (58.8)	NA
Prepregnancy body mass index, mean (SD)^a^	24.6 (5.0)	24.4 (4.4)	24.7 (5.3)
Race and ethnicity			
Hispanic	42 (9.0)	13 (8.1)	29 (9.5)
Non-Hispanic Black	69 (14.8)	14 (8.8)	55 (17.9)
Non-Hispanic White	312 (66.8)	115 (71.9)	197 (64.2)
Other^b^	44 (9.4)	18 (11.3)	26 (8.5)
Perceived body size at age 10 y			
Underweight	67 (15.2)	24 (15.7)	43 (15.0)
Average	322 (73.2)	113 (73.9)	209 (72.8)
Overweight	51 (11.6)	16 (10.5)	35 (12.2)
College graduate	344 (73.8)	132 (82.5)	212 (69.3)
Married or cohabitating	426 (91.4)	150 (93.8)	276 (90.2)
Annual household income >$70 000	285 (66.4)	114 (76.0)	171 (61.3)
Life’s Essential 8 scores			
Overall^c^	76.3 (12.2)	75.2 (12.4)	76.8 (12.0)
Behavioral domain^d^	76.5 (13.4)	76.8 (13.2)	76.4 (13.6)
Diet	47.8 (15.2)	47.9 (15.0)	47.6 (15.5)
Physical activity	83.0 (33.6)	85.5 (29.4)	81.7 (33.6)
Smoking status	91.4 (20.2)	90.5 (22.7)	91.9 (18.8)
Sleep health	83.9 (21.2)	83.7 (20.6)	84.0 (21.5)
Biomedical domain^e^	76.0 (17.5)	73.6 (18.0)	77.2 (17.2)
Body mass index	66.1 (34.0)	66.0 (32.5)	66.1 (34.7)
Blood pressure	79.9 (26.7)	80.5 (25.9)	79.6 (27.1)
Blood lipids^f^	67.9 (31.2)	61.3 (32.3)	71.4 (30.1)
Glycemia^g^	89.9 (19.4)	86.5 (23.1)	91.7 (16.9)
Blood biomarkers subdomain^h^	78.9 (19.2)	73.9 (20.7)	81.6 (17.9)

Abbreviation: NA, not applicable.

^a^ Body mass index is calculated as weight in kilograms divided by height in meters squared.

^b^ Other includes American Indian or Alaska Native, non-Hispanic Asian or Pacific Islander, and participants who endorsed more than 1 race.

^c^ Possible score range is 0 to 100, with higher scores indicating healthier outcomes.

^d^ Behavioral domain includes diet, physical activity, smoking status, and sleep duration.

^e^ Biomedical domain includes body mass index, blood pressure, blood lipids (non–high-density lipoprotein cholesterol), and glycemia.

^f^ Refers to calculated non–high-density lipoprotein cholesterol.

^g^ Refers to fasting glucose or hemoglobin A_1c_ plus diabetes status.

^h^ Blood subdomain includes blood lipids and glycemia.

Among those with an infertility history, the total LE8 score was 2.94 points lower (95% CI, −5.13 to −0.74 points), the biomedical domain score was 4.07 points lower (95% CI, −7.33 to −0.78 points), and the blood subdomain score was 5.98 points lower (95% CI, −9.71 to −2.26 points) vs those without such a history in fully adjusted models (Table 2). The point estimate also was lower for the behavioral domain score (β = −1.81; 95% CI, −4.28 to 0.66), although the 95% CI contained the null. In analyses of infertility with binary LE8 scores, our primary results were supported by decreased odds for high overall LE8 (odds ratio [OR], 0.56; 95% CI, 0.37 to 0.87), biomedical domain (OR, 0.56; 95% CI, 0.36 to 0.85), and blood subdomain (OR, 0.65; 95% CI, 0.43 to 0.99) scores (eTable 3 in Supplement 1).

**Table 2.** Associations Between History of Infertility and Life’s Essential 8 Scores in Project Viva at Midlife (N = 468)

Outcome at midlife	β (95% CI)^a^	
	Model 1	Model 2
Life’s Essential 8 overall score	−2.51 (−4.89 to −0.13)	−2.94 (−5.13 to −0.74)
Behavioral domain	−1.32 (−3.89 to 1.25)	−1.81 (−4.28 to 0.66)
Biomedical domain	−3.69 (−7.15 to −0.24)	−4.07 (−7.33 to −0.78)
Blood biomarkers subdomain	−6.17 (−9.86 to −2.48)	−5.98 (−9.71 to −2.26)

^a^ Values are β coefficients (95% CI) for multivariable linear regression adjusted for age at outcome (model 1) or adjusted for age at outcome, race and ethnicity, education at enrollment, household income at enrollment, age at menarche, and perceived body size at 10 years of age (model 2). Behavioral domain includes diet, physical activity, smoking status, and sleep. Biomedical domain includes body mass index, blood pressure, blood lipids, and glycemia. Blood subdomain includes blood lipids and glycemia.

To investigate the association of infertility with the LE8 biomedical domain, we conducted 2 secondary analyses. First, we also adjusted model 2 for the 4 scored behavioral domain components in a subanalysis (data not shown); biomedical domain point estimates were slightly attenuated (β = −3.66; 95% CI, −6.94 to −0.38). Second, we examined measured values for individual behavioral or biomedical domain components adjusted for the covariate set (Table 3). For these analyses, we used native units for each measure rather than the LE8 score. After multivariable adjustment, fasting glucose was 6.31 mg/dL (95% CI, 2.63 to 9.98 mg/dL; to convert glucose to millimoles per liter, multiply by 0.0555) and hemoglobin A_1c_ was 0.23% (95% CI, 0.11% to 0.34%; to convert hemoglobin A_1c_ to proportion of total hemoglobin, multiply by 0.01) higher among those with vs without an infertility history. The point estimate for the association of infertility with non-HDL cholesterol was in the hypothesized direction (7.66 mg/dL; 95% CI, −0.52 to 15.83 mg/dL; to convert cholesterol to millimoles per liter, multiply by 0.0259), although the 95% CI included the null. History of infertility was not associated with the other biomedical factors. No associations between infertility and behavioral components were observed (data not shown).

**Table 3.** Associations Between History of Infertility and Estimated Values for the 4 Components of Life’s Essential 8 Biomedical Domain in Project Viva at Midlife (N = 468)

Outcome at midlife	Mean (SD)	β (95% CI)^a^	
		Model 1	Model 2
Body mass index^b^	28.0 (6.5)	−0.10 (−1.36 to 1.17)	0.18 (−0.95 to 1.32)
Blood pressure, mm Hg			
Systolic	116.3 (14.4)	0.34 (−2.50 to 3.18)	0.62 (−2.15 to 3.39)
Diastolic	73.5 (10.9)	−0.63 (−2.77 to 1.52)	−0.45 (−2.54 to 1.65)
Blood lipids, mg/dL			
HDL cholesterol	68.8 (22.7)	−1.19 (−5.64 to 3.26)	−1.39 (−5.77 to 3.00)
Non-HDL cholesterol	140.0 (41.9)	7.90 (−0.18 to 15.99)	7.66 (−0.52 to 15.83)
Glycemia			
Fasting glucose, mg/dL	91.8 (18.1)	6.62 (2.96 to 10.29)	6.31 (2.63 to 9.98)
Hemoglobin A_1c_, %	5.3 (0.6)	0.23 (0.11 to 0.35)	0.23 (0.11 to 0.34)

Abbreviation: HDL, high-density lipoprotein.

SI conversion factors: To convert cholesterol to millimoles per liter, multiply by 0.0259; glucose to millimoles per liter, multiply by 0.0555; hemoglobin A_1c_ to proportion of total hemoglobin, multiply by 0.01.

^a^ Values are β coefficients (95% CI) for multivariable linear regression adjusted for age at outcome (model 1) and age at outcome, race and ethnicity (Hispanic, non-Hispanic Black, non-Hispanic White, or other, which includes American Indian or Alaska Native, non-Hispanic Asian or Pacific Islander, and participants who endorsed more than 1 race), age at menarche, perceived weight at age 10 years, and enrollment education (college graduate, yes/no) and household income (>$70 000, yes/no) (model 2).

^b^ Body mass index is calculated as weight in kilograms divided by height in meters squared.

In sensitivity analyses, we observed greater associations for those who experienced first infertility before age 35 years with overall LE8, biomedical domain, and blood subdomain than for those with first infertility at or after age 35 years (eTable 4 in Supplement 1). For example, compared with no experience of infertility, the estimated blood subdomain score among those with first infertility before age 35 years was 8.01 points lower (95% CI, −13.04 to −2.97 points) vs 4.32 points lower (95% CI, −8.97 to 0.33 points) among those with first infertility at or after age 35 years. No meaningful associations were observed between age at first infertility and the behavioral domain. In further sensitivity analyses (eTable 5 in Supplement 1), overall, point estimates were generally similar but slightly attenuated when excluding 31 participants with PCOS, slightly increased when excluding 26 participants with early menopause, and modestly increased when excluding all 189 participants who were past the menopausal transition at outcome assessment.

## Discussion

This cohort study examined the associations of history of infertility with CVH in female individuals at midlife via the AHA LE8. We observed rates of infertility similar to those in other studies^5,38,39^ and detected an association between infertility history and poorer CVH, including a 2.94-point lower overall LE8, 4.07-point lower biomedical domain, and 5.98-point lower blood biomarker subdomain scores. The associations with estimated blood components—non-HDL cholesterol, fasting glucose, and hemoglobin A_1c_—seemed to underlie the association between infertility and the biomedical domain, as well as the overall LE8 score. In sensitivity analyses, associations between infertility and LE8 domain scores were more pronounced for infertility experienced before age 35 years compared with age 35 years and later, except in the behavioral domain. These associations persisted after excluding individuals with PCOS, those with menopause occurring before age 45 years, or participants past the menopausal transition. A difference of 3% to 6% may be a modest change, but studies with the LE8 precursor, Life’s Simple 7*,* demonstrated that a 7% difference in scores was associated with differences in CVD outcomes,^20^ including lower risk by 18% for stroke and 20% for myocardial infarction.^40^ Although further investigations using the new LE8 are needed, our results suggest that infertility may be an important characteristic associated with long-term female CVH.

Our findings align with previous investigations that found history of infertility was associated with poorer CVH, although end points and CVD type differed by study. Infertility has been associated with increased risk among female individuals for any type of CVD,^41^ CVD events,^36^ CVD mortality,^42^ coronary heart disease (CHD),^43,44,45^ stroke,^43,44,46^ and heart failure.^43,47^ Several distinctions should be noted, however. In an analysis of the Nurses’ Health Study II, Wang et al^42^ reported that infertility was unrelated to premature CVD mortality in the overall sample, although individuals with infertility and no subsequent pregnancies had a 49% higher risk of CVD mortality (hazard ratio, 1.49; 95% CI, 1.06 to 2.10). Also in the Nurses’ Health Study II, Farland et al^45^ observed a 13% higher CHD risk with infertility history, but only with overweight or obesity; this association was preserved only with infertility occurring before age 25 years in age-stratified models. Compared with our analyses, this latter Nurses’ Health Study II study specifically examined associations of infertility with incident CHD and stroke at ages 53 to 70 years, whereas our investigation assessed associations with components of CVH. Studies have shown that higher CVH is associated with distinctly lower risk for CVD events.^20^ However, Farland et al^45^ did examine causes of infertility, particularly ovulatory dysfunction, which we could not assess. In addition, several studies used relatively young samples, with end points potentially before CVD outcomes could manifest. For example, among female individuals with a history of infertility, Gleason et al^36^ found 83% higher odds of cardiovascular events for individuals aged 20 to 59 years (mean, 40 years) from the National Health and Nutrition Examination Survey 2013 to 2014. Magnus and colleagues^41^ reported only slightly increased rates of CVD (hazard ratio, 1.07; 95% CI, 1.03 to 1.09) in female individuals aged 27 to 62 years (mean, 43 years) from the Norwegian Mother, Father and Child Cohort Study. Likewise, in the Swedish Medical Birth Registry (1983-2005), female individuals reporting 5 or more years of infertility (2.1%) with a subsequent childbirth had a 20% higher risk for hospitalization or death from CHD, stroke, or heart failure; however, no associations were observed with infertility duration less than 5 years.^43^

We are not aware of any other studies that have examined infertility in relation to health factors contained within the LE8, particularly components of the behavioral domain. It is not clear whether evidence exists for an association between infertility and later life behavioral factors. Evidence demonstrates direct associations of poorer quality diet,^48,49,50^ lower physical activity,^51,52^ shorter sleep duration,^52,53^ and smoking^54^ around the time of infertility. Although these components are important risk factors to assess, they may have temporal associations with infertility experiences, but may not affect later life behavioral factors unless potentially mediated by shared upstream risk factors (eg, mental or social health concerns).^55^

Our results indicate an important association of infertility with specific biomedical CVH risk factors, but supporting evidence is limited and conflicting. As related to self-reported infertility status and blood pressure, 2 studies^56,57^ found no difference at midlife (<49 years), and another study^58^ found no association with hypertension risk at age 45.5 years, except with infertility due to tubal disease (relative risk, 1.15; 95% CI, 1.01 to 1.31). As part of the Framingham Heart Study Third Generation and Omni Cohort 2 Exam 2 in 2008 to 2011,^56^ comprehensive examinations of CVD risk factors (ie, blood pressure, blood lipids, glycemia, BMI, and waist circumference) and their associations with female infertility found that BMI and waist circumference were the primary risk factors. This differed from our null finding for BMI. However, female infertility was also associated with lower HDL cholesterol (β, −3.23; 95% CI, −5.71 to −0.74),^56^ which diverges from our findings for HDL cholesterol but parallels the estimated increase in non-HDL cholesterol in our study. In addition, in a smaller study of 130 participants, 50% of whom had infertility, Verit et al^16^ detected higher triglycerides, total cholesterol, and low-density lipoprotein cholesterol alongside lower HDL cholesterol for individuals with previous infertility, but concluded that only triglycerides were associated with prior infertility. Within this setting of conflicting evidence, our findings support an association between history of infertility and non-HDL cholesterol, fasting glucose, and hemoglobin A_1c_ among female individuals in midlife. Furthermore, these associations appear to be greater with infertility occurring before age 35 years, highlighting the need for further investigation into CVH components

Disentangling the association between infertility and CVD remains challenging^19,59^ because infertility and CVH have an intimately interconnected and potentially confounded relationship (eg, PCOS, adiposity, insulin resistance, and metabolic syndrome may influence both outcomes).^60^ Ovulation disorders including PCOS account for approximately 25% of infertility cases, tubal disease accounts for 20%, endometriosis 5% to 10%,^61^ and diminished ovarian reserve accounts for 26% of infertility in US individuals seeking assisted reproductive technologies.^62^ Etiologic PCOS factors include insulin resistance and hyperglycemia that contribute to cardiovascular complications and atherosclerotic plaques.^63^ In addition, the precise causes of 30% of infertility cases cannot be identified,^61^ but 10% or more of unexplained female infertility cases may be due to genetic abnormalities.^64,65^

### Strengths and Limitations

Our study has many strengths. First, the use of a robust, longitudinal cohort with rich reproductive data and pertinent covariates, biomarker assessment, and long-standing follow-up provided a sturdy foundation to examine CVH. Second, the LE8 provides a targeted approach for continuous CVH assessment and monitoring for individuals and populations. Third, these findings suggest straightforward clinical applications. Results for LE8 overall, biomedical domain, and blood subdomain indicate associations with prior infertility; surveying individual reproductive histories for infertility may suggest earlier bloodwork assessment and ongoing screening, particularly in female individuals younger than 35 years, to provide a straightforward clinical evaluation of CVH. In addition, the AHA recommended using history of adverse pregnancy outcomes when assessing CVD risk and advised practitioner-directed guidance for lifestyle modification.^66^

We also acknowledge several limitations. At enrollment, Project Viva participants had insurance, health care, and resided in Eastern Massachusetts; thus, the results may be less generalizable to other populations.^21^ Indeed, we observed higher mean LE8 scores than the national mean (SE) of 68.10 (0.48).^67^ In addition, we cannot discount potential for selection bias in enrollment or attrition after approximately 18 years, or for nulliparous individuals not included per study design for whom we might expect greater associations.^68^ Furthermore, we could not assess causes of infertility, including those due to male factors. However, we did see similar results after excluding individuals with PCOS or menopause. Furthermore, the nature of Project Viva as a parous cohort cannot characterize the association between infertility and CVH for nulligravid and nulliparous individuals; however, these results highlight the importance of surveying reproductive history even in parous individuals.

## Conclusions

In this cohort study of parous individuals that examined associations of infertility with midlife CVH, infertility was associated with lower overall LE8, biomedical domain, and blood biomarker subdomain CVH scores compared with individuals without a history of infertility. These results were primarily associated with blood glucose, hemoglobin A_1c_, and non-HDL cholesterol levels. Our results provide additional weight to evidence suggesting inclusion of infertility history in assessing risk among female patients and demonstrate the importance of early identification and invocation of ongoing cardiovascular preventive strategies.

## Notes

Supplement 1. **eTable 1.** Characteristics of Originally Enrolled Project Viva Participants as Well as Those Included and Excluded From the Current Analyses **eTable 2.** Quantification of Cardiovascular Health Based on the American Heart Association’s *Life’s Essential 8* **eTable 3.** Associations Between History of Infertility and Life’s Essential 8 Score of High (≥80 Points) Versus Moderate/Low (<80 Points) in Project Viva at Midlife (n=468) **eTable 4.** Sensitivity Analysis of Associations Between Age in Years at First Report of Infertility and Life’s Essential 8 Scores in Project Viva at Midlife (n=466,^a^ 158 With Infertility) **eTable 5.** Sensitivity Analyses of Adjusted Associations Between Lifetime Infertility and Life’s Essential 8 Scores in Project Viva at Midlife Excluding Individuals With a History of PCOS (n=31), Early Menopause (n=26), or in Menopause (n=189) Compared to the Primary Sample

Supplement 2.
Data Sharing Statement
