# Supplementary material for: History of Infertility and Midlife Cardiovascular Health in Female Individuals
Source: JAMA Netw Open. 2024 Jan 5;7(1):e2350424. doi: 10.1001/jamanetworkopen.2023.50424 (PMC10770770; doi:10.1001/jamanetworkopen.2023.50424)
Supplement: Supplement 1. — eTable 1. Characteristics of Originally Enrolled Project Viva Participants as Well as Those Included and Excluded From the Current Analyses eTable 2. Quantification of Cardiovascular Health Based on the American Heart Association’s Life’s Essential 8 eTable 3. Associations Between History of Infertility and Life’s Essential 8 Score of High (≥80 Points) Versus Moderate/Low (<80 Points) in Project Viva at Midlife (n=468) eTable 4. Sensitivity Analysis of Associations Between Age in Years at First Report of Infertility and Life’s Essential 8 Scores in Project Viva at Midlife (n=466,a 158 With Infertility) eTable 5. Sensitivity Analyses of Adjusted Associations Between Lifetime Infertility and Life’s Essential 8 Scores in Project Viva at Midlife Excluding Individuals With a History of PCOS (n=31), Early Menopause (n=26), or in Menopause (n=189) Compared to the Primary Sample [file jamanetwopen-e2350424-s001.pdf]

Supplemental Online Content

Nichols AR, Rifas-Shiman SL, Switkowski KM, et al. History of infertility and midlife cardiovascular health in female individuals. *JAMA Netw Open*. 2024;7(1):e2350424. doi:10.1001/jamanetworkopen.2023.50424

eTable 1. Characteristics of Originally Enrolled Project Viva Participants as Well as Those Included and Excluded From the Current Analyses

eTable 2. Quantification of Cardiovascular Health Based on the American Heart Association's *Life's Essential 8*

eTable 3. Associations Between History of Infertility and Life's Essential 8 Score of High (≥80 Points) Versus Moderate/Low (<80 Points) in Project Viva at Midlife (n=468)

eTable 4. Sensitivity Analysis of Associations Between Age in Years at First Report of Infertility and Life's Essential 8 Scores in Project Viva at Midlife (n=466,[a] 158 With Infertility)

eTable 5. Sensitivity Analyses of Adjusted Associations Between Lifetime Infertility and Life's Essential 8 Scores in Project Viva at Midlife Excluding Individuals With a History of PCOS (n=31), Early Menopause (n=26), or in Menopause (n=189) Compared to the Primary Sample

This supplemental material has been provided by the authors to give readers additional information about their work.

eTable 1. Characteristics of Originally Enrolled Project Viva Participants as Well as Those Included and Excluded From the Current Analyses

**Characteristics**	**Overall** n=2100	**Included** n=468	**Excluded** n=1632
		**Mean (SD)**	
Age at enrollment, y	31.8 (5.2)	32.5 (5.2)	31.6 (5.2)
Pre-pregnancy BMI, kg/m^2	24.9 (5.0)	24.6 (5.01)	25.0 (5.7)
Race and Ethnicity, n(%)			
Hispanic	182 (8.8)	42 (9.0)	140 (8.7)
Non-Hispanic Black	346 (16.7)	69 (14.8)	277 (17.2)
Non-Hispanic White	1377 (66.3)	312 (66.8)	1065 (66.2)
Other[a]	172 (8.3)	44 (9.4)	128 (8.0)
Perceived body size at age 10y, n(%)			
Underweight	306 (17.3)	67 (15.2)	239 (18.0)
Average	1285 (72.8)	322 (73.2)	963 (72.7)
Overweight	174 (9.9)	51 (11.6)	123 (9.3)
College graduate, n(%)	1340 (64.6)	344 (73.8)	996 (61.9)
Married or cohabitating, n(%)	1895 (91.3)	426 (91.4)	1469 (91.3)
Household income >$70,000/y, n(%)	1124 (60.8)	285 (66.4)	839 (59.1)
Smoking status, ever, n(%)	654 (31.5)	125 (26.8)	529 (32.8)

[a]*Other includes non-Hispanic Asian.*

© 2024 Nichols AR et al. *JAMA Network Open.*

eTable 2. Quantification of Cardiovascular Health Based on the American Heart Association's *Life's Essential 8.*

Health Metric	Method of Measurement	Quantification of CVH Metric	
		Points	Measured Value
**Behavioral Domain**			
Dietary Intake	Calculated Healthy Eating Index (HEI) 2015 derived from self-reported 24-hr recall via the internet-based Automated Self-Administered Dietary Assessment Tool (ASA24)	100	≥95th percentile
		80	75th - 94th percentile
		50	50th - 74th percentile
		25	25th - 49th percentile
		0	<25th percentile
Physical Activity	Self-reported minutes/week of moderate or higher intensity activity	100	≥150 minutes
		90	120 - 149 minutes
		80	90 - 119 minutes
		60	60 - 89 minutes
		40	30 - 59 minutes
		20	1 - 29 minutes
		0	0 minutes
Smoking Status	Self-reported history of cigarette use, inhaled nicotine delivery system (e-cigarettes, vaping devices) use, or secondhand smoke exposure at home	100	Never
		50	Previous
		0	Current
		-20	Subtract 20 points if living with indoor smoker[a]
Sleep Health	Weighted average of self-reported hours of sleep/night for the prior month reported separately for week and weekend days	100	≥7 to <9 h/day
		90	≥9 to <10 h/day
		70	≥6 to <7 h/day
		40	≥5 to <6 or ≥10 h/day
		20	≥4 to <5 h/day
		0	<4 h/day
**Biomedical Domain**			
BMI	Weight (kg) measured with Tanita electronic scale (model TBF-3001, Arlington Heights, IL)<br>Height (m) measured with portable standing stadiometer (model SECA 213, Seca Worldwide, Hamburg, Germany)	100	<25 kg/m^2
		70	25.0 to 29.9 kg/m^2
		30	30.0 to 34.9 kg/m^2
		15	35.0 to 39.9 kg/m^2
		0	≥40.0 kg/m^2
Blood Pressure	Average of five blood pressure measurements with the OMRON Professional IntelliSense digital blood pressure monitor (Model HEM-907XL, OMRON Healthcare, Inc., Kyoto, Japan)	100	<120 & <80 mmHg
		75	≥120 to 129 & <80 mmHg
		50	≥130 to 139 or ≥80 to 89 mmHg
		25	≥140 to 159 or ≥90 to 99 mmHg
		0	≥160 or ≥100mmHG
		-20	Subtract 20 points if medication treated[a]
Blood Lipids[b]	Non-HDL cholesterol calculated as difference between plasma total cholesterol and HDL-cholesterol (mg/dL)	100	<130 mg/dL
		60	≥130 to 189 mg/dL
		40	≥160-189 mg/dL
		20	≥190 to 219 mg/dL
		0	≥220 mg/dL
		-20	Subtract 20 points if medication treated[a]
Glycemia[b]	Fasting blood glucose (FBG, mg/dL) or casual hemoglobin A1c (%), plus self-	100	No history of diabetes + FBG <100 or HbA1c <5.7%
		60	No history of diabetes + FBG 100-125 or HbA1c 5.7-6.4%
		40	Diabetes + HbA1c <7.0%

© 2024 Nichols AR et al. *JAMA Network Open.*

reported diabetes mellitus at the midlife visit	30	Diabetes + HbA1c 7.0 - 7.9%
	20	Diabetes + HbA1c 8.0 - 8.9%
	10	Diabetes + HbA1c 9.0 - 9.9%
	0	Diabetes + HbA1c ≥10.0.%

All metrics scored from 0 to 100, with a higher score indicating a healthier score. Overall LE8 score is an average of all eight component scores. [a]Only subtract points if score is not 0 points; [b]Components of the Blood Subdomain.

© 2024 Nichols AR et al. *JAMA Network Open.*

eTable 3. Associations Between History of Infertility and Life's Essential 8 Score of High (≥80 Points) Versus Moderate/Low (<80 Points) in Project Viva at Midlife (n=468)

	Model I		Model II	
**Outcome at Midlife**	**High (≥80) OR (95% CI)**	**Moderate/Low (<80) OR (95% CI)**	**High (≥80) OR (95% CI)**	**Moderate/Low (<80) OR (95% CI)**
***Life's Essential 8* Overall Score**	0.62 (0.42 to 0.94)	*Reference (1.0)*	0.56 (0.37 to 0.87)	*Reference (1.0)*
Behavioral Domain	0.77 (0.51 to 1.16)		0.70 (0.45 to 1.08)	
Biomedical Domain	0.61 (0.41 to 0.91)		0.56 (0.36 to 0.85)	
Blood Subdomain	0.63 (0.42 to 0.95)		0.65 (0.43 to 0.99)	

Values are Odds Ratios (95% CI) for multivariable logistic regression adjusted for: Model I: age at outcome. Model II: age at outcome, race and ethnicity (Hispanic, non-Hispanic Black, non-Hispanic White, other), age at menarche, perceived weight at 10y of age, and enrollment education (college graduate, yes/no) and household income (>$70,000, yes/no). Behavioral domain includes diet, physical activity, smoking status, sleep. Biomedical domain includes body mass index, blood lipids, glycemia, and blood pressure. Blood subdomain includes blood lipids and glycemia.

© 2024 Nichols AR et al. *JAMA Network Open.*

eTable 4. Sensitivity Analysis of Associations Between Age in Years at First Report of Infertility and Life's Essential 8 Scores in Project Viva at Midlife (n=466,[a] 158 With Infertility)

Outcome at Midlife	Model I<br>β (95% CI)	Model II<br>β (95% CI)
***Life's Essential 8* Overall Score**		
No infertility (n=308)	*Reference (0)*	
<35 years (n=64)	-3.28 (-6.53 to -0.03)	-3.67 (-6.66 to -0.68)
≥35 years (n=94)	-2.20 (-5.18 to 0.79)	-2.57 (-5.32 to 0.18)
**Behavioral Domain**		
No infertility (n=308)	*Reference (0)*	
<35 years (n=64)	-0.77 (-4.29 to 2.75)	-1.21 (-4.59 to 2.16)
≥35 years (n=94)	-2.13 (-5.36 to 1.10)	-2.60 (-5.70 to 0.50)
**Biomedical Domain**		
No infertility (n=308)	*Reference (0)*	
<35 years (n=64)	-5.80 (-10.51 to -1.08)	-6.13 (-10.60 to -1.66)
≥35 years (n=94)	-2.26 (-6.59 to 2.07)	-2.55 (-6.56 to 1.57)
**Blood Subdomain**		
No infertility (n=308)	*Reference (0)*	
<35 years (n=64)	-8.11 (-13.16 to -3.07)	-8.00 (-13.08 to -2.91)
≥35 years (n=94)	-4.56 (-9.20 to 0.08)	-4.31 (-8.99 to 0.37)

Values are β coefficients (95% CI) for multivariable linear regression for Model I adjusted for age at outcome and Model II adjusted for race and ethnicity, education at enrollment, household income at enrollment, age at menarche, and perceived body size at 10 years of age. Behavioral domain: diet, physical activity, smoking status, sleep. Biomedical domain: BMI, blood pressure, blood lipids, glycemia. Blood subdomain: blood lipids, glycemia. [a]Missing age at first pregnancy or infertility, n=2.

© 2024 Nichols AR et al. *JAMA Network Open.*

eTable 5. Sensitivity Analyses of Adjusted Associations Between Lifetime Infertility and Life's Essential 8 Scores in Project Viva at Midlife Excluding Individuals With a History of PCOS (n=31), Early Menopause (n=26), or in Menopause (n=189) Compared to the Primary Sample

Outcome at Midlife	Primary Sample (n=468) β (95% CI)	Excluding those with PCOS[a] (n=437) β (95% CI)	Excluding those with Early Menopause[b] (n=442) β (95% CI)	Excluding those in Menopause[c] (n=279) β (95% CI)
***Life's Essential 8* Overall Score**	-2.94 (-5.13 to -0.74)	-2.59 (-4.86 to -0.32)	-2.88 (-5.10 to -0.66)	-3.18 (-6.06 to -0.31)
Behavioral Domain	-1.81 (-4.28 to 0.66)	-1.80 (-4.41 to 0.81)	-1.37 (-3.82 to 1.08)	-2.12 (-5.51 to 1.27)
Biomedical Domain	-4.07 (-7.33 to -0.78)	-3.38 (-6.75 to -0.01)	-4.40 (-7.76 to -1.04)	-4.25 (-8.78 to 0.27)
Blood Subdomain	-5.98 (-9.71 to -2.26)	-4.70 (-8.50 to -0.89)	-6.16 (-9.99 to -2.32)	-6.20 (-11.26 to -1.14)

Values are β coefficients (95% CI) for multivariable linear regression adjusted for age at midlife visit, race and ethnicity, education at enrollment, household income at enrollment, age at menarche, and perceived weight at 10 years of age. Behavioral domain: diet, physical activity, smoking status, sleep. Biomedical domain: BMI, blood lipids, glycemia, and blood pressure. Blood subdomain: blood lipids and glycemia. [a]Infertility n=144; [b]Cessation of menstruation <45y, infertility n=153; [c]Cessation of menstruation >12mo, infertility=189.

© 2024 Nichols AR et al. *JAMA Network Open.*
