# Supplementary material for: History of Infertility and Midlife Cardiovascular Health in Female Individuals
Source: JAMA Netw Open. 2024 Jan 5;7(1):e2350424. doi: 10.1001/jamanetworkopen.2023.50424 (PMC10770770; doi:10.1001/jamanetworkopen.2023.50424)
Supplement: Supplement 2. — Data Sharing Statement [file jamanetwopen-e2350424-s002.pdf]

Data Sharing Statement

Nichols. History of Infertility and Midlife Cardiovascular Health in Female Individuals. *JAMA Netw Open*. Published January 05, 2024. doi:10.1001/jamanetworkopen.2023.50424

Data
Data available: No

Additional Information
Explanation for why data not available: Per Project Viva's data use and sharing policy, data are available upon reasonable request to project_viva@hphci.harvard.edu.
